# Blood-based microRNA profiling in patients with cardiac amyloidosis

**DOI:** 10.1371/journal.pone.0204235

**Published:** 2018-10-17

**Authors:** Anselm A. Derda, Angelika Pfanne, Christian Bär, Katharina Schimmel, Peter J. Kennel, Ke Xiao, P. Christian Schulze, Johann Bauersachs, Thomas Thum

**Affiliations:** 1 Institute of Molecular and Translational Therapeutic Strategies (IMTTS), IFB-Tx, Hannover Medical School, Hannover, Germany; 2 Department of Cardiology and Angiology, Hannover Medical School, Hannover, Germany; 3 Division of Cardiology, Columbia University Medical Center, New York, New York, United States of America; 4 Department of Medicine, Weill Cornell Medicine, New York, New York, United States of America; 5 Department of Internal Medicine I, Division of Cardiology, Pneumology, Angiology and Intensive Medical Care, University Hospital Jena, Friedrich-Schiller-University Jena, Germany; 6 Excellence Cluster REBIRTH, Hannover Medical School, Hannover, Germany; 7 Imperial College London, NHLI London, United Kingdom; University of Cincinnati College of Medicine, UNITED STATES

## Abstract

**Introduction:**

Amyloidosis is caused by dysregulation of protein folding resulting in systemic or organ specific amyloid aggregation. When affecting the heart, amyloidosis can cause severe heart failure, which is associated with a high morbidity and mortality. Different subtypes of cardiac amyloidosis exist e.g. transthyretin cardiac amyloidosis and senile cardiac amyloidosis. Today, diagnostics is primarily based on cardiac biopsies and no clinically used circulating blood-based biomarkers existing. Therefore, our aim was to identify circulating microRNAs in patients with different forms of amyloidosis.

**Methods:**

Blood was collected from healthy subjects (n = 10), patients with reduced ejection fraction (EF < 35%; n = 10), patients affected by transthyretin cardiac amyloidosis (n = 13) as well as senile cardiac amyloidosis (n = 11). After performing TaqMan array profiling, promising candidates, in particular miR-99a-5p, miR-122-5p, miR-27a-3p, miR-221-3p, miR-1180-3p, miR-155-5p, miR-339-3p, miR-574-3p, miR-342-3p and miR-329-3p were validated via quantitative real time PCR.

**Results:**

The validation experiments revealed a significant upregulation of miR-339-3p in patients affected with senile cardiac amyloidosis compared to controls. This corresponded to the array profiling results. In contrast, there was no deregulation in the other patient groups.

**Conclusion:**

MiR-339-3p was increased in blood of patients with senile cardiac amyloidosis. Therefore, miR-339-3p is a potential candidate as biomarker for senile cardiac amyloidosis in future studies. Larger patient cohorts should be investigated.

## Introduction

Amyloidosis is a rare and very heterogeneous disease, which can affect several human organs. Pathophysiological amyloidosis is caused by misfolding of non-soluble proteins, which deposit in an extracellular manner. When affecting the heart, cardiac amyloidosis can lead to to cardiac hypertrophy [1,2]. The most common type in the cardiac form is induced by light chain immunoglobulins (AL amyloidosis). In general, the AL type manifests systemically affecting also e.g. the liver or kidney. Another form of this disease is the transthyretin type amyloidosis also called ATTR amyloidosis. A different type affecting mainly men older than 70 years is the wild-type ATTR amyloidosis also named senile cardiac amyloidosis (SCA) [3]. For diagnosis of amyloidosis, cardiac biopsy and echocardiography are used. Until now, there is not any specific biomarker in blood known [3,4].

MicroRNAs (miR’s, miRNAs) are small ribonucleic acids about 20 nucleotides. As important regulators they orchestrate post-transcriptionally gene-expression and are therefore involved in cell differentiation processes [5]. In cardiac diseases they are important players in pathologic processes like e.g. cardiac fibrosis and hypertrophy [6]. Therefore, microRNAs can be used as therapeutic targets. More and above, microRNAs circulating in the blood are used as biomarkers for example in Takotsubo syndrome or Hypertrophic cardiomyopathy [7,8]. The aim of our study was to find specific blood-based microRNAs in serum of patients with different forms of cardiac amyloidosis.

## Materials and methods

### Study population

All patients including control and heart failure individuals were recruited at the Columbia University Medical Center in New York. All patients gave written informed consent to participate in the study, which was conducted in accordance with the protocol approved by the Institutional Review Board at Columbia University Medical Center. The diagnosis of different types of amyloidosis was defined by myocardial biopsy and echocardiography. In the heart failure group (HF) only patients with an ejection fraction (EF) < 35% and without amyloidosis or other genetic diseases were included.

### RNA isolation from patient blood samples

To obtain liquid supernatant by separating the corpuscular components, the serum samples were centrifuged for 10 minutes at 2000 x g at room temperature. Afterwards the supernatant was stored at -80°C. The miRNeasy Mini Kit (Qiagen) was utilized according to instructions for the RNA isolation. For subsequent normalization synthetic *Caenorhabditis elegans* miR-39 (5 μl of 1 fmol/μl) was added as spike-in control during RNA isolation to the Qiazol/chloroform/plasma mixture [8].

### TaqMan ArrayCard microRNA screening

For performing a microRNA expression profile isolated RNA of single individuals were pooled within their groups. After preamplification, TaqMan gene expression array cards– 384-well microfluidic cards (ThermoFisher SCIENTIFIC) were used according to manufacturer’s instructions. 753 different reactions of microRNA were measured. For analyzing array data we used -ddCT method.

### Validation of microRNA candidates using realtime PCR

TaqMan MicroRNA Reverse Transcription Kit (Applied Biosystems) was used according to developer’s instructions to transcribe the isolated RNA of each blood sample to their complementary DNA (cDNA). For validation the microRNAs miR-99a-5p, miR-122-5p, miR-27a-3p, miR-221-3p, miR-1180-3p, miR-155-5p, miR-339-3p, miR-574-3p, miR-342-3p and miR-329-3p were chosen. By using TaqMan MicroRNA assays (Applied Biosystems) the microRNAs were amplified with quantitative realtime PCR (qrt-PCR). Afterwards expression results were normalized to cel-miR-39. qPCR was performed on a Viia7 Real-Time PCR system (Thermo Fisher Scientific) (15 min at 95°C, 45 cycles of 15 sec at 95°C and 1 min at 60°C). The QuantStudioTM Real-Time PCR software (v1.1) was used to determine quantification cycle. Ct was defined as the fractional cycle number at which the fluorescence exceeded a given threshold. Relative quantification was performed using the 2-dCt method, where dCt = Ct[miRNA]—Ct[cel-miR-39].

### Statistical analysis

For statistical analysis and graphical illustration of demographics and expression data Graph Pad Prism 6 was utilized. For analyzing the regulation of miRNAs between the different patient groups and control based on Ct-values Kruskal-Wallis test (Nonparametric One-way ANOVA multi comparison test) were used. Graphs were illustrated as scatter plots with standard deviation. For array card analysis p-values < 0,05 defined significance using a two-sided t-test.

## Results

### Clinical characteristics

In this study, 10 controls, 10 heart failure patients, 13 TTR mutation patients and 11 senile cardiac amyloidosis patients were included. There were no statistical significant differences in age of controls, heart failure and TTR group but the patients affected with senile cardiac amyloidosis were significantly older than control and TTR group. Comparing the gender, in all groups more men than women were included (Table 1). There was no difference in BMI. In the heart failure group we included more patients with diabetes mellitus but this was without statistical significance. Comparing cholesterol, in particular LDL and HDL cholesterol, there was also no difference (Table 2).

**Table 1:** Patient groups.

				
	Control	HF	TTR	SCA
number	10	10	13	11
age [y] ± SD	62.70 ± 6.45	70.50 ± 6.08	67.54 ± 8.31	78.45 ± 4.57
gender	f = 3; m = 7	f = 0; m = 10	f = 4; m = 9	f = 0; m = 11

HF = heart failure; TTR = transthyretin; SCA = senile cardiac amyloidosis; y = years; SD = standard deviation; f = female; m = male

**Table 2:** Demographics.

				
	HF	TTR	SCA	HF vs. TTR vs. SCA
BMI [kg/sq m) ± SD	26.18 ± 2.46	27.42 ± 5.20	25.24 ± 4.13	ns
Diabetes mellitus [%]	50.00% (n = 5)	15.38% (n = 2)	27.27% (n = 2)	ns
LVEF [%] ± SD	26.00 ± 5.35	36.25 ± 16.67	41.18 ± 15.14	ns
BNP [pg/ml] ± SD	341.4 ± 336.9	775.3 ± 821.6	818.4 ± 862.0	ns
Hct [%] ± SD	39.59 ± 4.17	40.18 ± 5.01	39.27 ± 6.90	ns
HDL [mg/dl] ± SD	37.33 ± 8.31	45.33 ± 16.67	51.33 ± 13.94	ns
LDL [mg/dl] ± SD	73.22 ± 24.19	81.89 ± 41.75	82.17 ± 43.61	ns

HF = heart failure; TTR = transthyretin; SCA = senile cardiac amyloidosis; BMI = body mass index; LVEF = left ventricular ejection fraction; BNP = Brain natriuretic peptide; Hct = Haematocrit; ns = not significant; H/LDL = High/Low Density Protein

### MicroRNA-Array results

**Fig 1:**
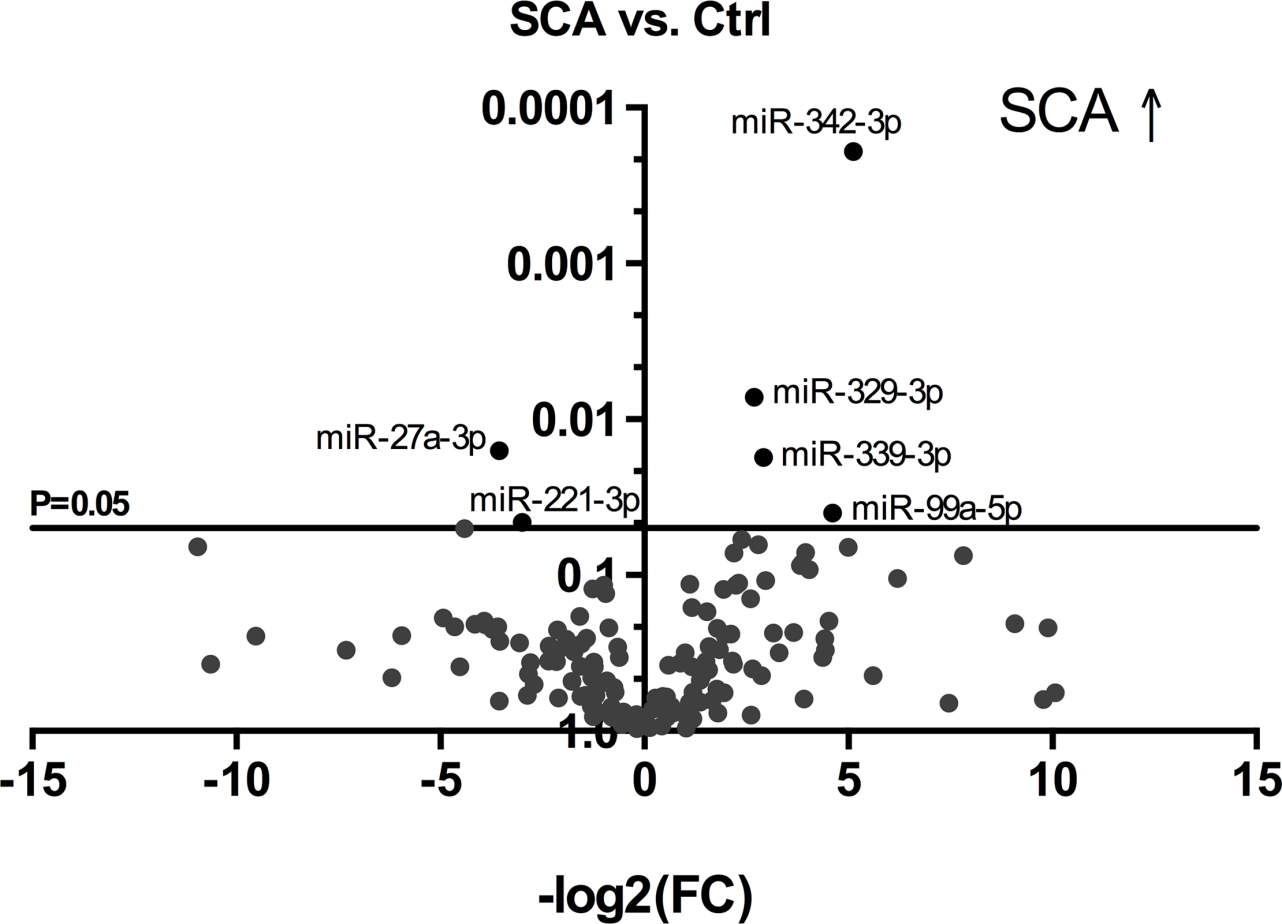
Volcano plot comparing SCA to control. X-axis = -log2 of Fold Change (FC), Y-axis = p-value; SCA = senile cardiac amyloidosis. SCA ↑ = SCA upregulation.

**Fig 2:**
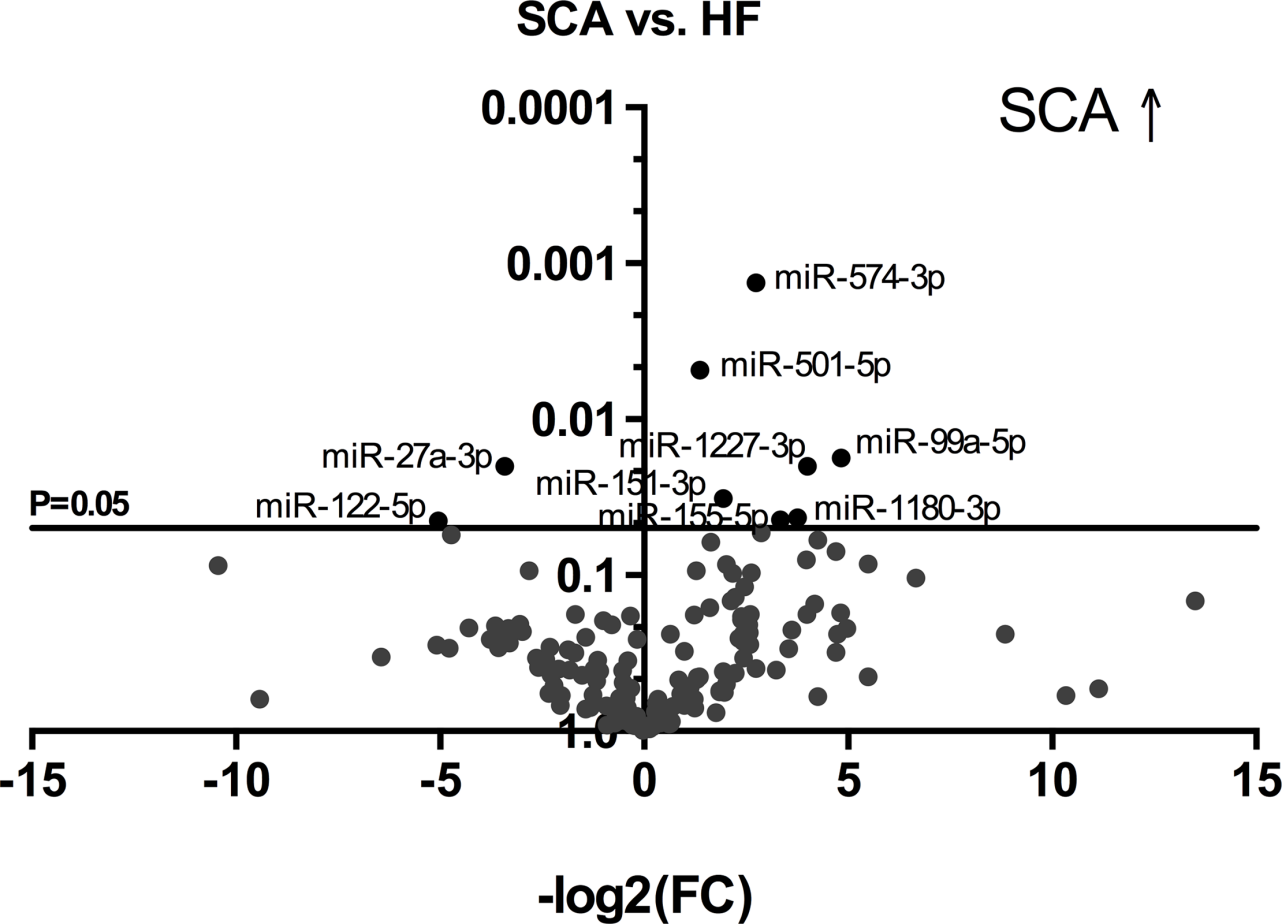
Volcano plot comparing SCA to HF. X-axis = -log2 of Fold Change (FC), Y-axis = p-value; SCA = senile cardiac amyloidosis; HF = heart failure. SCA ↑ = SCA upregulation.

**Fig 3:**
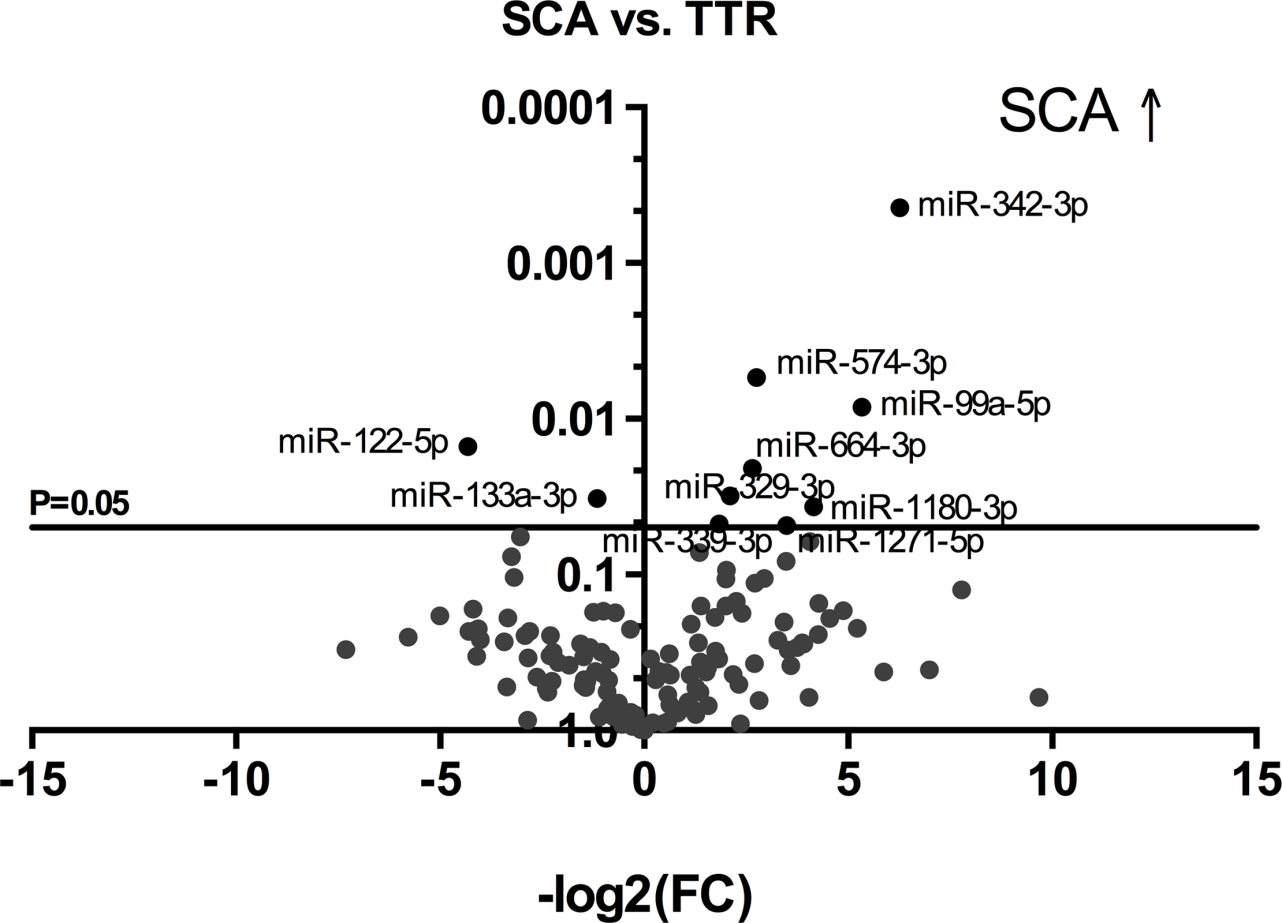
Volcano plot comparing SCA to TTR. X-axis = -log2 of Fold Change (FC), Y-axis = p-value; SCA = senile cardiac amyloidosis; TTR = transthyretin. SCA ↑ = SCA upregulation.

Comparing the control group to SCA patients, miR-329-3p, miR-342-3p, miR-339-3p and miR-99a-5p were significantly upregulated in SCA. In contrast, miR-27a-3p and miR-221-3p were decreased (Fig 1). When comparing the heart failure group with SCA group there was a significant upregulation of miR-574-3p, miR-501-5p, miR-99a-5p, miR-1227-3p, miR-151-3p, miR-1180-3p and miR-155-5p. Similar to the control group miR-122-5p was decreased as well as miR-27a-3p (Fig 2). Nevertheless, while comparing TTR group and SCA patients the miRNAs miR-342-3p, miR-574-3p, miR-99a-5p, miR-664-3p, miR-1180-3p, miR-1271-5p, miR-329-3p and miR-339-3p were significantly increased in SCA group. Whereas, miR-122-5p and miR-133a-3p were downregulated (Fig 3). Based on these results the microRNAs miR-99a-5p, miR-122-5p, miR-27a-3p, miR-221-3p, miR-1180-3p, miR-155-5p, miR-339-3p, miR-574-3p, miR-342-3p and miR-329-3p were chosen for validation.

### Validation data

**Fig 4:**
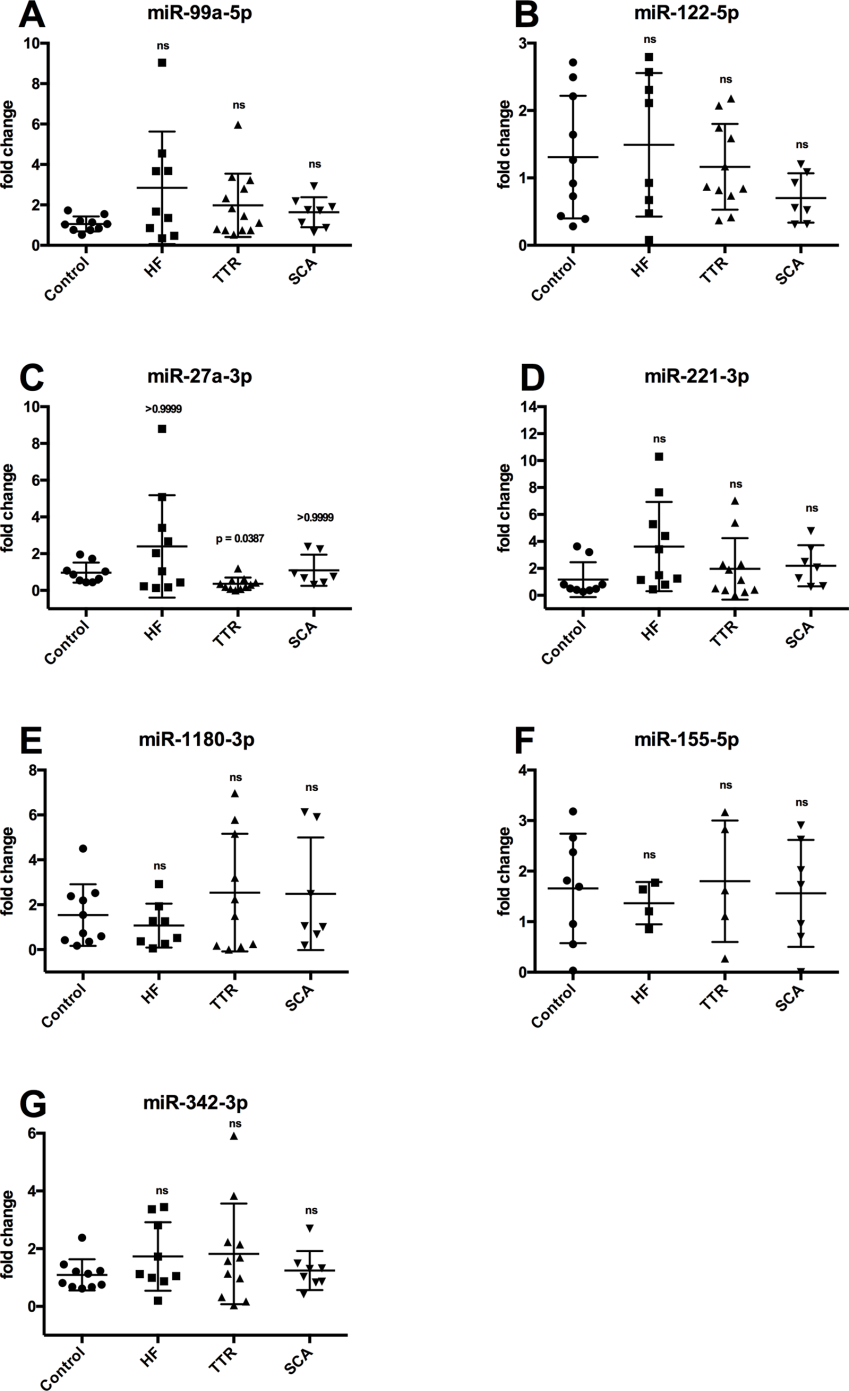
Validation. microRNA expression in controls, heart failure (HF) patients, transthyretin (TTR) group and senile cardiac amyloidosis (SCA). ns = not significant.

**Fig 5:**
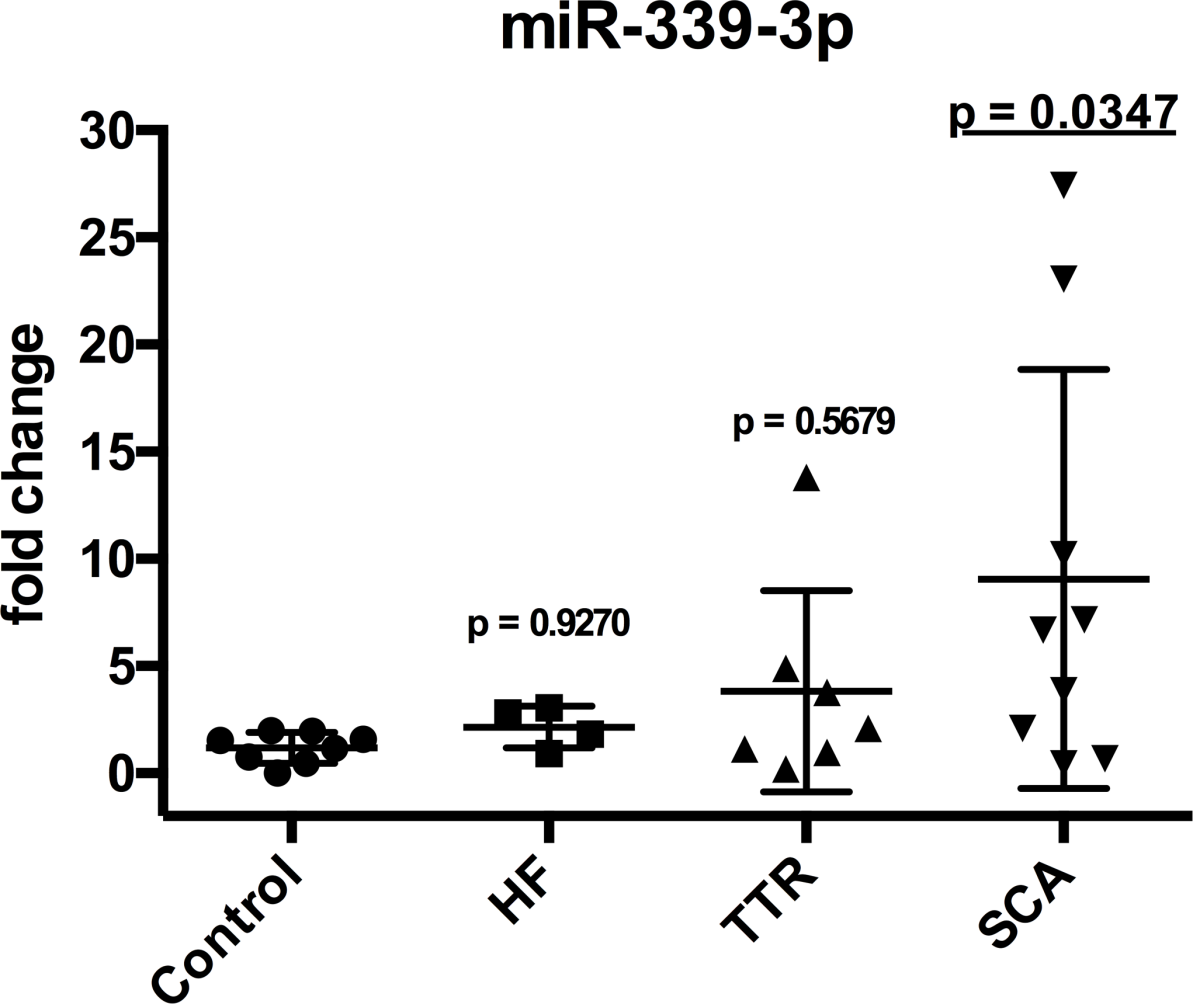
Validation. microRNA miR-339-3p expression in controls, heart failure (HF) patients, transthyretin (TTR) group and senile cardiac amyloidosis (SCA).

Looking at the validated microRNA selection, there was no dysregulation of the circulating miR-99a-5p and miR-122-5p between the different groups (Fig 4A and 4B). Beside it, the circulating expression of miR-27a was not increased in any group, but significantly decreased in TTR patients compared to control (Fig 4C). There were also no differences in circulating levels of the microRNAs miR-221-3p, miR-1180-3p, miR-155-5p and miR-342-3p (Fig 4D, 4E, 4F and 4G). In addition, levels of miR-339-3p were significantly increased in patients with senile cardiac amyloidosis whereas not in the other patient groups (Fig 5). Primer sequences of validated microRNAs are shown in Table 3.

**Table 3:** Mature miR sequences.

	
microRNA	sequence
hsa-miR-27a-3p	UUCACAGUGGCUAAGUUCCGC
hsa-miR-99a-5p	AACCCGUAGAUCCGAUCUUGUG
hsa-miR-122-5p	UGGAGUGUGACAAUGGUGUUUG
hsa-miR-155-5p	UUAAUGCUAAUCGUGAUAGGGGUU
hsa-miR-221-3p	AGCUACAUUGUCUGCUGGGUUUC
hsa-miR-329-3p	AACACACCUGGUUAACCUCUUU
hsa-miR-339-3p	UGAGCGCCUCGACGACAGAGCCG
hsa-miR-342-3p	UCUCACACAGAAAUCGCACCCGU
hsa-miR-574-3p	CACGCUCAUGCACACACCCACA
hsa-miR-1180-3p	UUUCCGGCUCGCGUGGGUGUGU

## Discussion

In this study, we performed a microRNA assay in samples derived from patients with different forms of cardiac amyloidosis and validated promising biomarker candidates to distinguish different forms of amyloidosis, heart failure patients as well as healthy individuals. Up to date, there is no other study showing the regulation of microRNAs in cardiac amyloidosis. In array profiling experiments, we found 10 candidates being deregulated in TTR amyloidosis and senile cardiac amyloidosis.

MicroRNA miR-99a, which was upregulated in array profiling in patients with SCA, was recently shown as tumor suppressor in lung cancer working together with oncogenic proteins EMR2 and E2F2 [9]. In validation experiments, this miRNA failed as possible biomarker for cardiac amyloidosis. Also, miR-574-3p and miR-329 was detected only in a very low number of patient samples. Moreover, miR-122, which was previously described as marker of neurological damage in blood of patients after cardiac arrest [10], did not show any significant regulation in patients with cardiac amyloidosis. Further, miR-155 also failed in our validation cohort as potential biomarker for cardiac amyloidosis. In heart diseases, miR-155 is described as a promoter of cardiac hypertrophy leading to heart failure [11]. Interestingly, we found that miR-339-3p was significantly upregulated in both array data and validation analysis compared to control samples. Until today, miR-339-3p has not been described as a specific circulating biomarker in any heart disease. There is also no previously reported association between miR-339-3p and amyloidosis. It is known that miR-339-3p functions as a suppressor in melanoma by targeting the oncogene MCL1 [12]. Besides melanoma, miR-339-3p is also described as inhibitor for colorectal cancer tumor growth in in-vitro experiments [13]. Thus, miR-339-3p was mainly known in the cancer field. However, our work suggests that miR-339-3p will be a promising candidate as clinical biomarker for senile cardiac amyloidosis. The exact pathophysiological mechanisms of the origin and secretion of miR-339-3p into the blood in cardiac amyloidosis remains unclear and needs to be explored in future studies. Although, miR-221 was described as promoter of cardiac hypertrophy [14], it was not dysregulated in our validation experiments. Also expression levels of miR-27a described e.g. as anti-hypertrophic in rat cardiomyocytes [15] and miR-1180-3p were not significantly dysregulated in any of our patient groups. Interestingly, miR-342-3p being described as decreased in acute heart failure did not show any dysregulation either in our heart failure group or in cardiac amyloidosis groups [16,17].

In this study are also limitations. The unbalanced age of the senile cardiac amyloidosis group is most likely due to the incidence of the disease in higher ages. Another limitation is the small sample size within the different groups owing to the relative rareness of cardiac amyloidosis and therefore the small patient population. In addition, our study did not reveal the pathomechanism behind the upregulation of miR-339-3p in patients with senile cardiac amyloidosis.

## Conclusion

In conclusion, miR-339-3p was increased in the plasma of patients with senile cardiac amyloidosis as identified by array profiling and validated by qPCR. Thus, miR-339-3p is a potential candidate biomarker for senile cardiac amyloidosis. Larger cohorts should be investigated in the future.

## Supporting information

S1 Array Supplement (XLSX)
